# A comparative genomics study on the effect of individual amino acids on ribosome stalling

**DOI:** 10.1186/1471-2164-16-S10-S5

**Published:** 2015-10-02

**Authors:** Renana Sabi, Tamir Tuller

**Affiliations:** 1 Department of Biomedical Engineering, Tel Aviv University (TAU), Tel Aviv, Israel; 2 The Sagol School of Neuroscience, Tel-Aviv University (TAU), Tel-Aviv, Israel

**Keywords:** mRNA translation, nascent peptide, ribosome stalling, ribosomal exit tunnel, comparative ribosome profiling, transcript evolution

## Abstract

**Background:**

During protein synthesis, the nascent peptide chain emerges from the ribosome through the ribosomal exit tunnel. Biochemical interactions between the nascent peptide and the tunnel may stall the ribosome movement and thus affect the expression level of the protein being synthesized. Earlier studies focused on one model organism (*S. cerevisiae*), have suggested that certain amino acid sequences may be responsible for ribosome stalling; however, the stalling effect at the individual amino acid level across many organisms has not yet been quantified.

**Results:**

By analyzing multiple ribosome profiling datasets from different organisms (including prokaryotes and eukaryotes), we report for the first time the organism-specific amino acids that significantly lead to ribosome stalling. We show that the identity of the stalling amino acids vary across the tree of life. In agreement with previous studies, we observed a remarkable stalling signal of proline and arginine in *S. cerevisiae*. In addition, our analysis supports the conjecture that the stalling effect of positively charged amino acids is not universal and that in certain conditions, negative charge may also induce ribosome stalling. Finally, we show that the beginning part of the tunnel tends to undergo more interactions with the translated amino acids than other positions along the tunnel.

**Conclusions:**

The reported results support the conjecture that the ribosomal exit tunnel interacts with various amino acids and that the nature of these interactions varies among different organisms. Our findings should contribute towards better understanding of transcript and proteomic evolution and translation elongation regulation.

## Background

mRNAs translation is a fundamental intracellular process which occurs in all living organisms. Translation elongation is an iterative stage of translation in which the ribosome scans the mRNA sequence and decodes it into a specific protein by adding one amino acid at the time to the growing peptide chain. It has been suggested that the speed by which ribosomes progress along the mRNA is affected by different local features of the coding sequence. One determinant of the translation elongation speed is the identity of the codon at the P-site; it has been suggested that the codon decoding rate is influenced by several factors related to the P-site, including: the cellular concentration of the paired tRNA [1-6]; the efficiency of the codon-anticodon pairing which occurs non-optimally for wobble base pairing [7-9] and the efficiency of incorporation of the decoded amino acid into the polypeptide which is mainly poor in the case of proline [10-12]. Other coding sequence features thought to slow down ribosomes include: the folding energy of the mRNA sequence downstream from the ribosomal P-site [13-16]; the identity of the tRNA at the A-site [17]; and the charge of the amino acids in the exit tunnel [16,18,19].

The Ribosomal Exit Tunnel (*RET*) is the site through which nascent peptides leave the ribosome during translation. The non-uniform biochemical characteristics of the tunnel allow it to play an important role in affecting translation rates and protein folding rather than being a passive conduit for the nascent polypeptide. First, the overall electrostatic potential of the *RET *is negative and varies in magnitude along the tunnel [19-23]; thus, it was suggested that a nascent peptide that contains charged amino acids may undergo electrostatic interaction with the exit tunnel [19]. Second, the diameter of the tunnel varies between 10A^0 ^and 20A^0 ^[24-27]; thus, the interaction between that nascent peptide and the exit tunnel may also be dictated by geometrical constraints. Although the expansion in diameter enables the partial folding of the translated peptide [28], the cramped dimensions of the tunnel prohibit a folding of whole protein domains and only tertiary/secondary structures of small segments are allowed [29].

Evidences of ribosome pausing mediated by nascent peptide have been manifested in several studies [30-36]. These studies, however, either conducted a small scale experiment or focused on one organism only.

The development of the ribosome profiling technique has significantly broaden the comprehension of in vivo translation by enabling the detection of the momentary positions of ribosomes along the transcripts at nucleotide resolution [37]. During the past few years, the high throughput quantitative data obtained by ribosome profiling experiments has been widely used to study gene translation [10,16,18,38-51].

Specifically, ribosome profiling data was used to show that ribosome stalling is induced in response to the presence of certain amino acid [10,16,18]. Specifically, it has been suggested that positively charged amino acids are implicated in transient ribosomal pauses by interacting with the negatively charged exit tunnel [16,18,19]. A more recent study of Artieri and Fraiser [10], on the other hand, emphasized the possibility that the incorporation of proline into the nascent peptide has the major effect on ribosome stalling.

In order to investigate the organism-specific influence of each individual amino acid on substantial ribosome stalling, we performed a large scale analysis based on multiple ribosome profiling datasets of 9 organisms including eukaryotes (*H.sapiens, C.elegans, S.cerevisiae, S.pombe, A.thaliana, P.falciparum, D.melanogaster, M.musculus*) and bacteria (*C.crescentus*).

## Results

**Figure 1:**
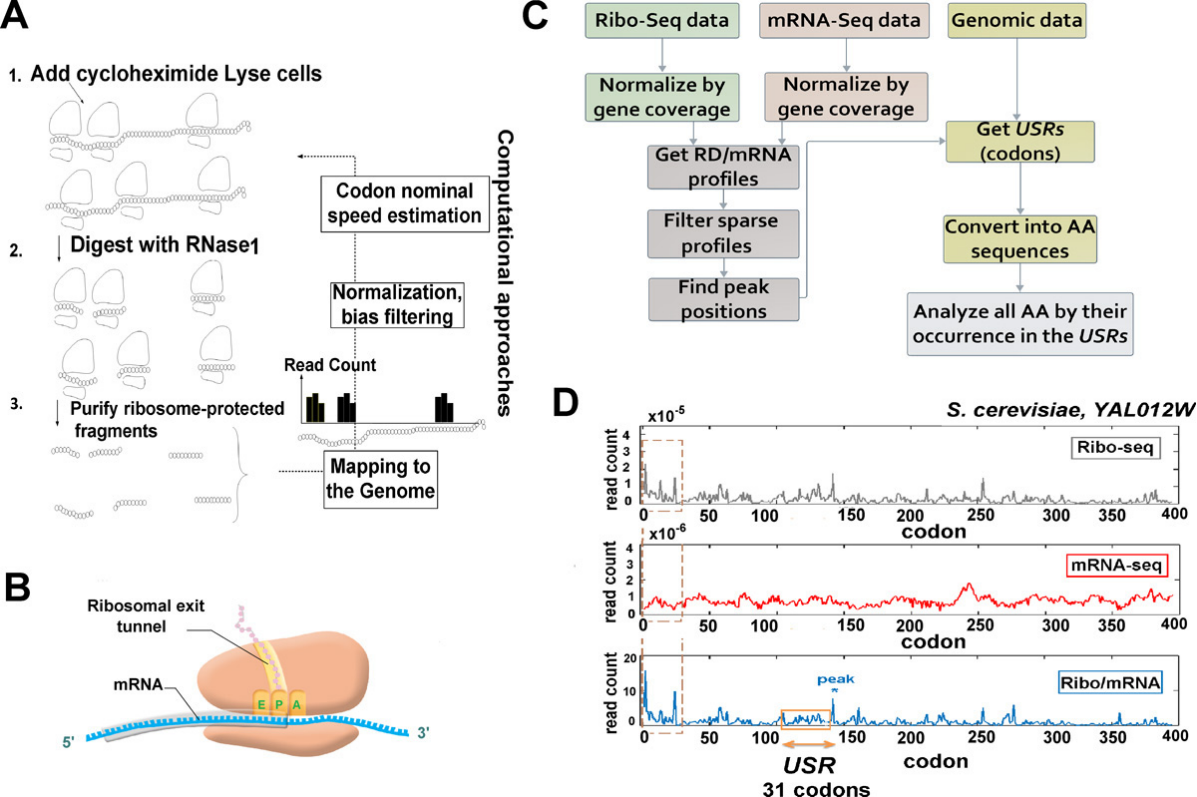
**General description of the approach described in the study**. **(A)** The major steps of the ribosomal profiling approach: 1) Cells are treated with cycloheximide, for example, to arrest translation; 2) Ribosomes are fixed and ribosome-protected RNA fragments are recovered; 3) After processing and reverse-transcription, these are sequenced, mapped and used to derive a ribosomal density profile. **(B) **An illustration of the ribosome and the exit tunnel during translation elongation. The sequence of codons upstream from the ribosomal A-site (shaded in gray) represents the amino acid sequence that occupies the exit tunnel while the codon at the P-site is being translated (depicted by pink circles). **(C) **The general steps of the approach described in this study: Ribo-seq and mRNA-seq profiles are normalized by the average gene coverage; new profiles are generated based on the ratio between ribo-seq reads and mRNA reads; normalized profiles with sparse coverage are filtered; peak positions in RD/mRNA are extracted; the codons *USR *of each peak is converted into amino acid sequence (denoted as AA) and each amino acid is analyzed based on its frequency in all *USRs *(see specific details in the Methods). **(D) **An example of ribo-seq, mRNA-seq and RD/mRNA profiles obtained from gene YAL012W in *S. cerevisiae*. The profiles were generated based on all *S. cerevisiae *datasets (see the Methods section: *Merging all datasets of the organism into one aggregate*). Positions along each profile represent the location of the ribosomal A-site. The first 20 codons (marked by a dashed brown frame) are excluded from the analysis (details in the Methods section: *Data filtering*). The 31 codons upstream from the peak are the Upstream Stalling Region of codons corresponding to the amino acid sequence in the exit tunnel.

Ribosome profiling experiments include the following major stages (Figure 1A): cells are treated with cycloheximide (for example) to arrest translating ribosomes; then, RNA fragments protected by ribosomes from RNases are isolated and processed for high-throughput sequencing, resulting in reads of ribosomes protected footprints. As slowly decoded codons are covered by ribosomes for a larger amount of time, they tend to create higher amount of protected fragments, in comparison to faster decoded codons on the same transcript. Finally, using a computational method, the obtained sequenced footprints are mapped to the genome of the analyzed organism creating for each gene a ribosomal footprints read count profile. This profile will be referred here as a *RD *profile for Ribosomal Density.

In this work, we aim at understanding whether extreme ribosomal stalling occurs at a specific codon is affected by an interaction between the *RET *and the amino acids encoded by the codons upstream from the pause (Figure 1B). To this end, we use ribo-seq and mRNA-seq data to generate normalized profiles of RD/mRNA and extract peak positions in each normalized profile (Figure 1C). These positions presumably represent the positions along the mRNA where ribosomes have been significantly stalled (see details in the Methods). In the next step, we define for each peak the corresponding Upstream Stalling Region (*USR*) which is the sequence of amino acids encoded by the codons upstream from the peak. These amino acids occupy the *RET *while the codon at the peak position is being translated. Specifically, since the length of peptide required to fill the tunnel is approximately 31 amino acids [52], we have focused on the 31 amino acids before each peak (Figure 1D).

The folding of the nascent peptide inside the exit tunnel [28] and additional factors may alter the distance of a specific amino acid in the tunnel from the P-site during the translation process [21,53,54]; thus, we have decided to use measures that are based on the enrichment of different amino acids in the *USR *instead of constraining the amino acids to appear at a specific position relative to the P-site.

### The organism-specific stalling effect of each amino acid

**Figure 2:**
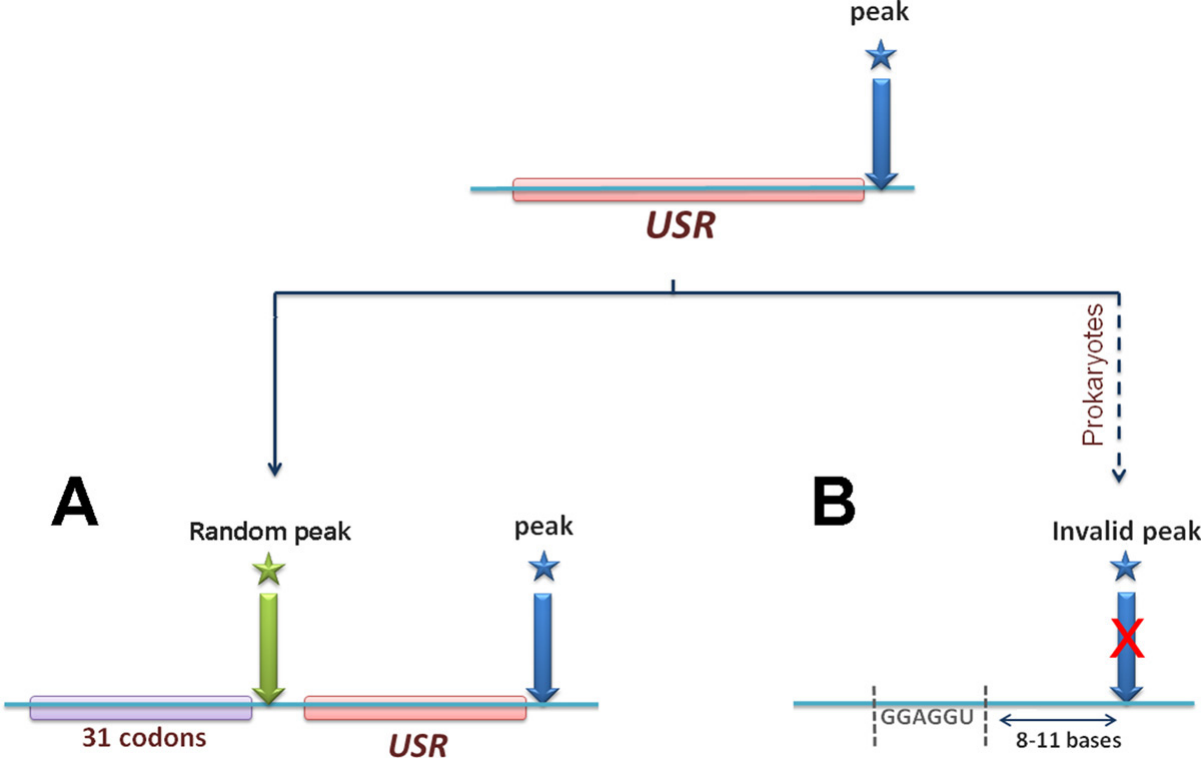
**Tests for identifying stalling amino acids**. The position of peak in the RD/mRNA profile is marked by a blue arrow. The *USR *corresponding to the peak is the upstream sequence of 31 codons which codes for the 31 amino acid that occupy the tunnel when ribosomes stall at the peak position. **(A) **In the first test, the frequency of each amino acid upstream of real peak positions is compared with its frequency in the 31 codons upstream of random positions. The number of randomly drawn positions per profile is equal to the number of real peaks in the original profile (see details in the Methods). **(B) **The additional test is performed for bacteria. In case a sequence that resembles the *SD *sequence was observed 8-11 bases upstream from the peak position, the peak was excluded from the analysis.

At the first step, we determined the enrichment of each amino acid in the *USRs *based on the following test: we calculated the probability to observe the amino acid in the real *USRs*; then, we calculated the probability to observe the amino acid in randomized ribosome profiling with similar properties as the original data (see details in the Methods section: *Quantifying the enrichment of each amino acid in the USRs*). Finally, based on the real and randomized ribosomal profiling data, we calculated a p-value which determines the extent to which each amino acid tends to occupy the *RET *while a codon at a highly stalled position is being translated (Figure 2A). For the bacteria, we performed one additional test (Figure 2B) to show that the reported results cannot be explained by the fact that hybridization between the prokaryotic ribosomal RNA and sequences that resemble the Shine-Dalgarno (SD) sequence can also cause pauses [50,55]; this phenomenon was controlled by filtering peaks that appear downstream from such sequences (see details in the Methods section: *Controlling for translational pausing driven by Shine-Dalgarno-like sequences*).

**Figure 3:**
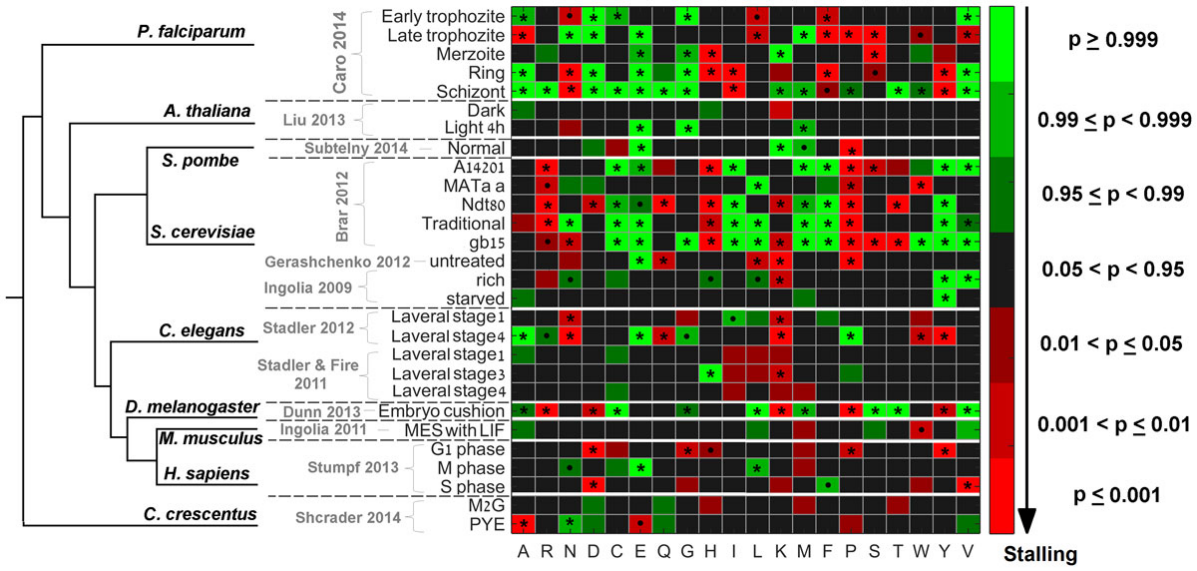
**Dataset-specific classification of the 20 amino acids**. Each amino acid was classified as significantly stalling (red), significantly non-stalling (green) or insignificant (black) according to the frequency of its codons in the *USRs*. All analyzed datasets are listed to the left. A color bar with the different significance levels is provided to the right. Stalling amino acids that passed FDR at the 0.05 level are marked with asterisk and those that passed FDR at the 0.1 level are marked by black dots. Thick horizontal white lines are plotted to separate the different organisms which are ordered in accordance with their evolutionary tree based on iTOL [76,77].

For each dataset, we classified each amino acid in one of three possible classifications based on the output of the randomized *USR *test: If the test turned out to be significant, the amino acid was classified as 'overrepresented', meaning that the analysis supports the hypothesis that this amino acid tends to appear upstream of peaks more than expected by the null model (this may suggest that the amino acid contributes to the ribosomal stalling via its interaction with the tunnel). If the test turned out to be significant in the opposite direction (*i.e.*, the probability of observing the amino acid in *USRs *was significantly *smaller *than in random regions), the amino acid was classified as 'underrepresented'. In case the test turned out to be insignificant at the 0.05 level, the amino acid was classified as 'not significant'. The classification of the 20 amino acids for each of the analyzed datasets appears in Figure 3.

As can be seen in Figure 3, our analysis suggests that the amino acids which significantly tend to occupy the exit tunnel when ribosomes stall, are organism (or condition)-specific.

Two remarkable stalling signals were produced by Proline (P) and Arginine (R) in *S. cerevisiae*, a finding that is well supported by the study of Artieri and Fraser [10]. In addition, our results suggest that Proline has also a stalling effect in more organisms including *S. pombe, D. melanogaster, H. sapiens *(G1 phase), *P. falciparum *(Late trophozite) and *C. crescentus *(PYE). Another new prominent stalling effect was observed for Lysine (K) in all datasets of *C.elegnas*.

The negatively charged glutamic acid (E) was not found to be significantly stalling in any eukaryotic dataset. Moreover, it exhibited a significant signal of non-stalling in 6 of the 8 eukaryotes. Aspartic acid (D), the second negatively charged amino acid exhibits a stalling signal in specific datasets from 3 organisms (*S. cerevisiae, D. melanogaster and H. sapiens*).

### Identifying the regions in the exit tunnel that tend to interact with the growing peptide

**Figure 4:**
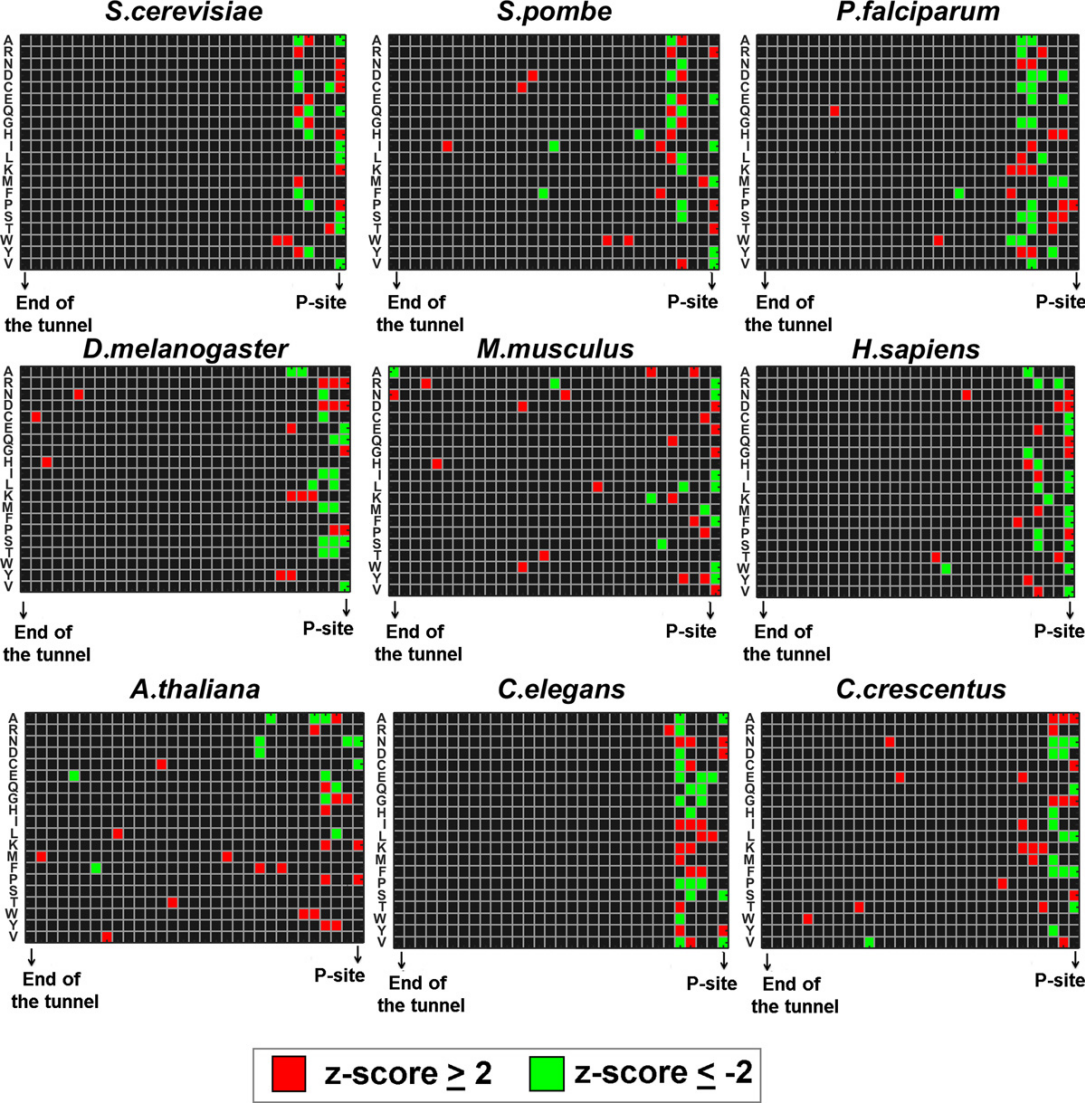
**The distribution of amino acids along the tunnel when ribosomes stall**. The position-specific probabilities were calculated for the 31 positions in the tunnel (based on the *USRs*). Results are presented per organism based on an aggregate that merges all analyzed datasets of the organism (see details in the Methods section: *Merging all datasets of the organism into one aggregate*). The probabilities were standardized to have a mean of zero per amino acid. We defined a square to be red/green if the probability to observe the amino acid in the corresponding position is significantly higher/lower than other positions in the tunnel.

Since the biochemical, geometrical and electrostatic properties of the tunnel varies along its length, specific regions in the tunnel may have higher potential to induce interactions with certain amino acids. In order to identify such regions, we calculated the probability for each amino acid to occupy a specific position along the length of the exit tunnel when the ribosome stalls (*i.e*. upstream from peak positions). The resultant position-specific probabilities for each organism are presented in Figure 4.

As can be seen, for most of the amino acids, the positions with the most extreme probabilities tend to be concentrated in the part of the tunnel that is close to the P-site (~5 amino acids in length). This might suggest that in most cases this part of the tunnel tends to undergo more interactions with the translated amino acids than other positions in the tunnel. It can also be seen that proline, tends to specifically appear in the ribosomal P-site in 6 of the 9 organism, in line with previous studies [10-12].

### Charged amino acids do not contribute to ribosome stalling in all organisms

**Figure 5:**
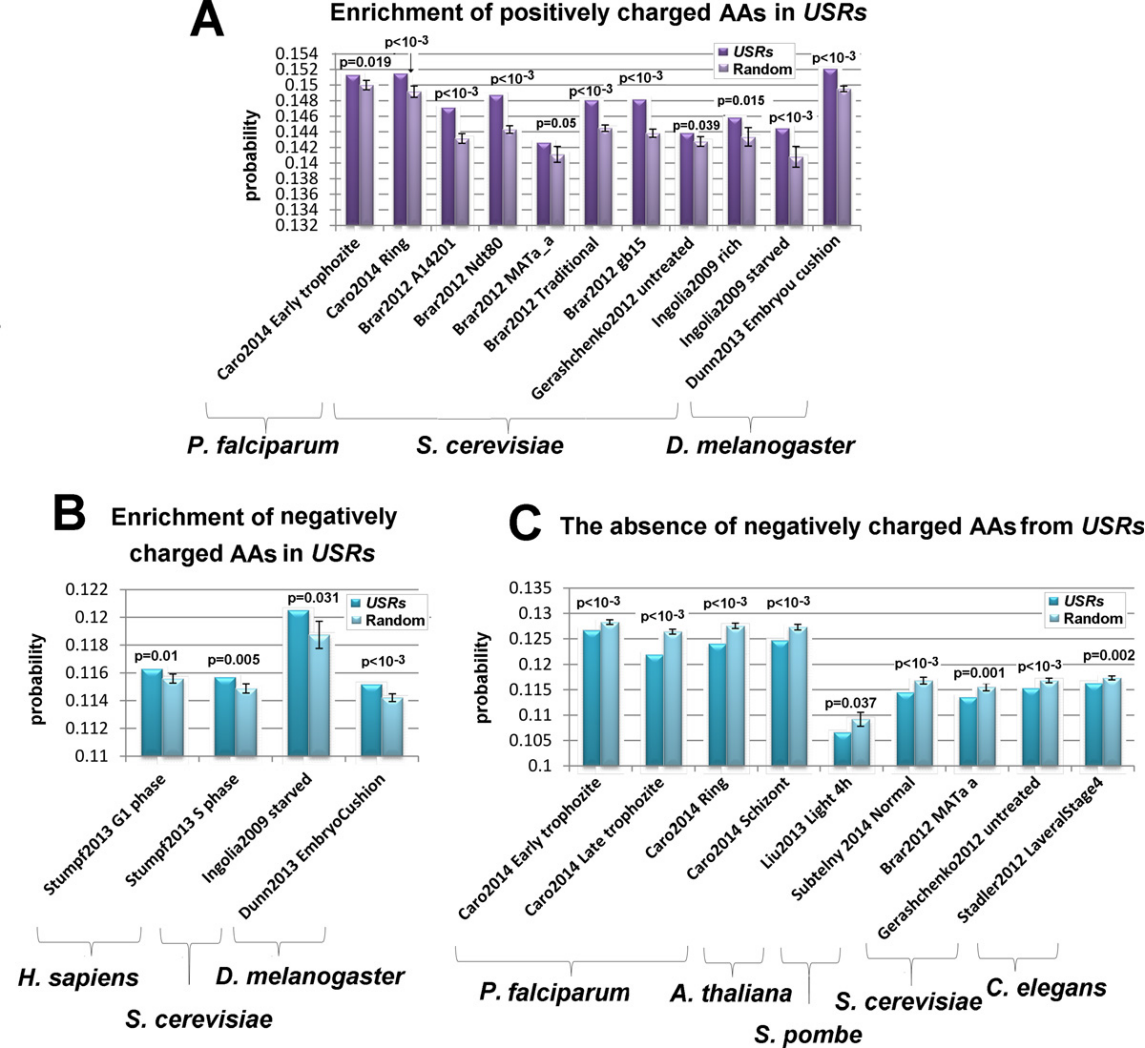
**Enrichment of charged amino acids in *USRs***. The probability to observe a charged amino acid in the real *USRs *is compared to the probability to observe it before random peak positions (details in the Methods section: *Quantifying the enrichment of charged amino acids in USRs*). The probabilities in random are average values over all randomizations. Standard deviations and p-values are also presented. **(A) **The probability to observe a positively charged amino acid in real and random *USRs *for the 11 cases that exhibited a significant p-value (2 datasets of *P. falciparum*; 8 datasets of *S. cerevisiae*; and one of *D. melanogaster*). **(B) **The probability to observe a *negatively *charged amino acid in real and random *USRs *for the 4 cases that exhibited a significant p-value (2 datasets of *H. sapiens*, one datasets of *S. cerevisiae *and one datasets of *D. melanogaster*) **(C) **The probability to observe a *negatively *charged amino acid in real and random *USRs *for the 9 cases where the probability was significantly higher *in random *than in the real *USRs *(4 datasets of *P. falciparum*; one dataset of *A. thaliana*, one dataset of *S. pombe*; 2 datasets of *S. cerevisiae *and one of *C. elegans*).

Earlier studies have suggested that charged amino acids tend to interact with the exit tunnel and thus, contribute to ribosomal stalling [16,18,19,56]. Our analysis demonstrates that indeed in a few cases, the *USRs *tend to be enriched with charged amino acids (Figure 5). In order to understand whether the factor for the stalling is specifically the charge or other property of the amino acid, we tested the composite effect of charged amino acids on ribosome stalling. Similarly to the tests described in Figure 2, the frequency of occurrence of the charged amino acids was compared between real and randomized *USRs *(See details in the Methods section : *Quantifying the enrichment of charged amino acids in USRs)*.

As can be seen in Figure 5, enrichment of positively charged amino acids among *USRs *was observed in 11 datasets from 3 eukaryotic organisms. Enrichment of *negatively *charged amino acids among *USRs *was less common and was observed only in 4 datasets from 3 organisms. On the contrary, in 5 organisms the probability to observe negatively charged amino acid before peaks was significantly higher in random (Figure 5C); this may suggest that negatively charged amino acids can prevent the halting of the ribosome. We found no cases of greater enrichment in random for the positively charged amino acids.

## Discussion

Our analysis identified nascent single amino acids that with high confidence contribute to ribosome stalling. The approach taken here to detect these amino acids is based on strict definitions and includes important controls on the analyzed genes such as control for amino acids bias and for possible experimental/protocol biases. In addition, we performed for the first time multi-organismal study of this topic which includes the analysis of both prokaryotes and eukaryotes.

The statistical tests performed here are based on the enrichment of amino acids *upstream *from the ribosomal P-site, thus, features such as mRNA folding strength which tends to slow ribosomes down when it occurs downstream from the P-site cannot trivially explain our results. In addition, previous studies (*e.g*. [18,39]) have suggested that the effect of rare codons on ribosome stalling tends to be less extreme than the effect of the interaction between the ribosomal exit tunnel and the nascent chain; thus, we also believe that the reported results cannot be trivially explained by the use of rare/non-efficient codons (see Additional file 1 for analysis supporting this point).

It is important to mention that currently the biases arise from the ribosome profiling approach and the effect of different protocols are not completely understood [57,58]. Much effort was spent here to consider these possible biases by 1) excluding from the analysis the first 20 codons which are known to be biased [37,44,57]; 2) filter low-coverage profiles; 3) normalizing each profile by its mean coverage to account for coverage differences [10] 4) normalizing ribo-seq data by mRNA-seq data to account for shared biases between the two fractions [10] 5) analyzing many datasets corresponding to a few different experimental conditions; 6) analyzing and comparing nine organisms (including eukaryotes and prokaryotes); 7) excluding pauses which might have been caused by SD sequences that hybridize with the prokaryotic ribosomal RNA [50,55]. Taken together, the reported results are based on a very conservative approach.

One of the major conclusions is related to the relation between positively charged amino acids and ribosome stalling. Previous studies have suggested that in *S. cerevisiae *positively charged amino acids play a role in ribosome stalling. Our analysis supports this conjecture in *S. cerevisiae *and also in specific datasets from *D. melanogaster *and *P. falciparum*. Therefore, our study suggests that the relation between amino acids charge and ribosomal halting is not universal.

In addition, our analysis suggests that not only positively charged amino acids interact with the *RET*. Specifically, we show that negatively charged amino acids tend to halt the ribosome via interactions with the exit tunnel in *S. cerevisiae *(Ingolia *et al*. 2009, starved condition growth [37]), *D. melanogaster *(Dunn *et al*. 2013, Embryos cushion [59]) and *H. sapiens *(Stumpf *et al*. 2013, G1 and S phase of HeLa cells [60]). Since the *RET *is negatively charged [19-21] it makes sense that it may undergo interactions with both positively and negatively charged amino acids. Furthermore, interestingly our analysis suggests that in some cases the negatively charged amino acids may prevent stalling; this may be related to charge cancellation with possible positively charged amino acids that co-appear in proximity in the exit tunnel.

Although we discuss the stalling effect of each amino acid on ribosome stalling, we do not claim that the stalling is manipulated by a specific mechanism. In fact, the explanation regarding the exact type of interaction between these amino acids and the ribosome and the reason they differ across the tree of life is an open question for future studies.

The reported results support the conjecture that the amino acids composition of the nascent peptide affects the ribosomal translation speed and might even cause ribosomal arrest. Thus, this finding suggests a complex interaction between the protein co-translational folding, protein amino acid content and ribosomal elongation speed: the translated amino acids affect translation speed which may affect protein folding. Thus, we believe that there is a co-evolution among these variables.

The fact that different stalling amino acids were reported for the different analyzed organisms may suggest that the biochemical properties of the exit tunnel vary along the tree of life and/or in different conditions [61-64]. This finding also provides important insights about heterologous gene expression: the expression of the same protein in different organisms may affect its translation rate simply due to the different nature of the interactions between the protein amino acids and the ribosomal exit tunnels in new organisms. This fact can explain why the topic of heterologous gene expression is often very challenging and why synonymous manipulation on the protein alone is not always sufficient for solving problems in this field.

Finally, as a future research it would be interesting to generalize the results reported here by estimating the effect of short peptides and sets of amino acids (not necessarily neighbor amino acids) in the *RET *on ribosomal halting. For example, since stalling peptides interfere with translation, they are expected to be selected against to improve translational efficiency. Thus, it would be interesting to examine the relation between the stalling effect of these peptides and their representation in the proteome. However, this mission is statistically challenging due to the exponential increase in the number of sets of amino acids compositions with more than one amino acid.

## Conclusions

In this work, we performed a multiple ribosome profiling datasets analysis to understand the effect of different amino acid on ribosome stalling. The reported results support a few conjectures: various amino acids interact with the ribosomal exit tunnel; the nature of these interactions is organism/condition specific and the nascent translated peptide tends to have more interactions with the beginning of the exit tunnel (close to the p-site).

## Methods

### Coding Sequences Data

Coding sequences of all analyzed organisms were retrieved from the *UCSC *genome browser (http://genome-euro.ucsc.edu).

### Ribo-seq and mRNA-seq data

Ribo-seq and mRNA-seq data used in this study are based on the following experiments: Ingolia *et al*. 2009 [37], Brar *et al*. 2012 [46] and Gerashchenko *et al*. 2012 [43] for *S. cerevisiae*; Subtelny *et al*. 2014 [65] for *S. pombe*; Stadler and Fire 2011 [66] and Stadler *et al*. 2012 [67] for *C. elegans*; Ingolia *et al*. 2011 [40] for *M. musculus*; Stumpf *et al*. 2013 [60] for *H. sapiens*; Dunn *et al*. 2013 [59] for *D. melanogaster*; Caro *et al*. 2014 [68] for *P. falciparum*; Liu *et al*. 2013 [69] for *A. thaliana *and Schrader *et al*. 2014 [70] for *C. crescentus*. Ribosomal footprints reads of each experiment have been uniquely mapped to the corresponding genome by Michel *et al*. 2014 [71] and were retrieved from the GWIPS-viz database (http://gwips.ucc.ie).

### Mapping ribosomal footprints to genomic positions

The specific genomic position assigned to each read represents the location of the ribosomal A-site on the mRNA. In GWIPS-viz, the genomic coordinate of each read has been determined differently for eukaryotes and prokaryotes. For the eukaryotic fragments, in which the 5′ end of the footprint is sufficient to carry the positional information [37,40], an off-set of 15 nucleotides from the 5' end of the fragment was used. Prokaryotic fragments, in contrast, varied between 25 and 40 nucleotides in length, mostly as a result of the specificity of micrococcal nuclease and thus, a weighted centered approach implemented by Oh *et al*., 2011 [47] was used to indicate the putative location of the ribosomal A-site. Specifically, 12 nucleotides were trimmed from each end of the prokaryotic fragment and the remaining residues were given a score of 1/N, where N equals the number of positions leftover after discarding the 5' and 3' ends, and blurring the signal across the central residues.

### Data filtering

The density of ribosome footprints is significantly elevated in the beginning of the gene due to a combination of biological phenomena and biases [37,40,44,72]. Thus, the first 20 codons were excluded from all aspects of the analysis described in this study. In addition, to account for biases related to sparse coverage, genes' profiles with fewer than 40 percent non-zero read counts were further filtered.

### RD peaks definition

While ribosome profiling data is given at nucleotide resolution, our analysis is based on codons. Thus, we averaged the read counts at each three bases corresponding to codons triplets to get the density profile at codon resolution. Then, in order to define peak positions in a given profile, we calculated the average read count (excluding zeros and the first 20 codons) and consider positions that exceed the average by 4 standard deviations as peaks. This definition was chosen empirically by ensuring that the total *USRs *in a protein sequence will cover at the most 20 percent of its length (otherwise it is not possible to randomize these sequences).

### Accounting for biases in mRNA-seq data and coverage differences

Theoretically, mRNA-seq read counts along a specific transcript should be uniformly distributed. In practice, due to various biases, this is not the case and the read counts obtained by mRNA-seq differ along the transcript (yet with small deviation from the average read count of the transcript relatively to ribo-seq read counts).

Recently Artieri and Fraser [10], established a robust methodology to account for such biases which includes the normalization of ribo-seq data by mRNA-seq data. Similarly to their approach, we first scaled each profile (ribo and mRNA) by the gene coverage and then, calculated the RD/mRNA ratio for each codon.

We compared the results obtained by the normalized data (*i.e*. the RD/mRNA ratio) with those obtained by the *RD *data without the normalization. The amino acids classifications based on the non-normalized data (Additional File 2), clearly produces more false signals compared to those obtained by the normalized data (Figure 3).

### The robustness of the reported results to a stricter threshold of coverage data

A general problem in large scale analyses is finding a work point where the signal to noise ratio is optimal. Specifically, in the case of ribosome profiling data, there is a tradeoff between a high-coverage demand (which is necessary for understanding the global behavior across the transcript) and a large number of genes (which strengths the statistical power of the reported signal). In our case, we analyzed only genes with at least 40% non-zeros read counts (Methods). In order to benchmark this definition, we compared the results obtained by this definition against a stricter one (for example a threshold of 60% non-zero read counts in each profile). We observed no cases where amino acid changed the direction of significance, demonstrating the robustness of the reported results. The classified amino acids for the stricter threshold are presented in Additional File 3.

### The effect of rare codons on the reported results

In order to show that the reported results cannot be trivially explained by the use of rare codons, we calculated Spearman's rank correlation coefficient between the probability that each codon occupies the P-site at peak positions and its corresponding tRNA adaptation index (*tAI*). The *tAI *is a widely used measure of the adaptation of codons to the tRNA pool of the organism [2], thus, it provides an information regarding the nominal translation rate of codons. The tRNA gene copy numbers used for the *tAI *calculation were retrieved from the Genomic tRNA database [73] (http://gtrnadb.ucsc.edu). The correlations for each of the nine analyzed organisms are presented in Additional File 1.

### Merging all datasets of the organism into one aggregate

An aggregate that is based on all analyzed datasets of the organism was generated in two steps: First, to cancel the effect of different coverage between datasets, we sum the read counts over all profiles to get the total number of read counts in the experiment; then, we normalized the read counts in each dataset by its total number of reads. Second, we averaged the normalized profiles of each gene to get the final aggregate. This was done for both, the ribo-seq and the mRNA-seq data. Finally, the normalization by gene coverage and mRNA-seq discussed in the previous section was performed on the aggregate dataset. Aggregate datasets were used to generate Figure 1D, 5 and Additional File 1.

### Controlling for translational pausing driven by Shine-Dalgarno-like sequences

It is known that in bacteria hexanucleotide sequences that resemble Shine-Dalgarno (*SD*) features within coding sequences can cause translational pausing due to hybridization between the mRNA and the 16S ribosomal RNA of the ribosome [50]. We have defined a *SD *sequence as a hexanucleotide sequence which contains up to one substitution relative to the canonical *SD *(GGAGGU). Specifically, the optimal spacing between the 3' end of the anti-*SD *sequence and the ribosomal A-site is 8-11 nucleotides [74]. Thus, we have excluded peaks that contain a *SD *sequence 8, 9, or 11 nucleotides upstream from the peak position.

### Quantifying the enrichment of charged amino acids in *USRs*

In order to understand whether positively charged amino acids (Lysine, arginine and histidine) tend to stall the ribosome via interaction with the *RET*, we quantified their tendency to appear before *RD *peaks (*i.e.*, in *USRs*). Traversing all peaks in all genes, we gave each peak a binary score: +1, if at least one positively charged amino acid (any of the three) appears in the *USR *of the peak and 0 if none of the three is observed. Finally, we summed up all peaks to get the total score (statistics) of the positively charged amino acids for the entire *USRs *($score_{pos\text{\_}AA,USRs}$); then, these value were normalized by the number of peaks to get the empirical probability. In order to quantify the significance of the score, we generated a null model by randomly draw the positions of the peaks maintaining the number of peaks in each gene. Random *USRs *are equivalently the 31 amino acids sequences upstream from each random peak position. The score of the positively charged amino acids ($score_{pos\text{\_}AA,random}$) was calculated based on the random peaks. The process was repeated 1000 times.

The empirical p-value that determines the extent to which the frequency of occurrence of positively charged amino acids is higher in real *USRs *than in random was calculated by:

$$p_{pos\text{\_}AA,USRs}=\frac{number\;of\;times\left(score_{pos_{AA},random}\geq score_{pos_{AA},USRs}\right)}{1000}$$

$p_{pos\text{\_}AA,USRs}<0.05$ indicates a significant enrichment of positively charged amino acids in *USRs*.

Similarly, we defined a p-value for the enrichment of negatively charged amino acids (Glutamate or aspartic acid) in *USRs*. A score of +1 was given to a peak if at least one negatively charged amino acid (any of the two) appears in the *USR *of the peak. The empirical p-value was calculated by:

$$p_{neg\text{\_}AA,USRs}=\frac{number\;of\;times\left(score_{neg_{AA},random}\geq score_{negAA,USRs}\right)}{1000}$$

For each type of charge, the scores do not change if both positive/negative charge appear in the *USR *(*e.g.*, if we perform the test for the negatively charged amino acid, we will give a score of +1 to every *USR *which included any negatively charged amino acid even if a positively charged amino acid also appears). Allowing both types of charge is based on the following rational: First, the statistical power of the test is higher (since we do not omit *USRs*). Second, we believe that the interactions between positive or negative amino acid and the exit tunnel occur is certain region of the exit tunnel; since these regions may change in different conditions/organisms or during the translation of a certain mRNA, a positively/negatively charged amino acid may affect the ribosomal movement even if there is an additional positively/negatively charged amino acid in the *USR*. Finally, this definition is more conservative since the calculated p-value might be higher (less significant) in cases of charge cancellation (*i.e*., when the positive and negative charge in the *USR *cancel each other effect).

### Quantifying the enrichment of each amino acid in the *USRs*

Similarly to the approach described in the previous paragraph, we have quantified the tendency of each single amino acid to stall the ribosome based on its occurrence in the *USRs*. For each amino acid, we traverse all peaks in all genes and assign each peak a binary score: +1 if the amino acid in question appears in the *USR *corresponding to the peak and 0 if it is not. Finally, we sum over all peaks to get $score_{AA\left(i\right),USRs}$, the total score of the amino acid for the entire *USRs *of the proteome. For the null model we randomly draw the positions of the peaks, while maintaining the number of peaks in each profile to be identical to the actual profile, and calculate $score_{AA\left(i\right),random}$ to each amino acid. The process was repeated 1000 times.

The p-value for the *i*-th amino acid is defined by:

$$p_{AA\left(i\right),USRs}=\frac{number\;of\;times\;\left(score_{AA\left(i\right),random}\geq score_{AA\left(i\right),USRs}\right)}{1000}$$

$p_{AA\left(i\right),USRs}<0.05$ indicates a significant enrichment of the *i*-th amino acid in the *USRs*. To control the False Discovery Rate (FDR), we performed a multiple testing correction on the resultant p-values (based on the Benjamini-Hochberg procedure [75]).

## Abbreviations

RET: Ribosomal Exit Tunnel; RD: Ribosomal Density; USR: Upstream Stalling Region; AA: Amino Acid; SD: Shine-Dalgarno; FDR: False Discovery Rate; tAI: tRNA Adaptation Index.

## Competing interests

The authors declare that they have no competing interests.

## Authors' contributions

Conceived and designed the experiments: RS TT. Analyzed the data: RS TT. Wrote the paper: RS TT.

## Supplementary Material

Additional File 1 **The correlation between *tAI *and P-site occupation probability at peak positions**. The results are presented per organism based on an aggregate that merges all analyzed datasets of the organism (see details in the Methods section: *Merging all datasets of the organism into one aggregate*). The probability at the x-axis represents the probability that each of the 61 sense codons occupies the P-site at peak positions. Spearman's rank correlation coefficient (rho) and a corresponding p-value (p) are to the upper right hand corner of each figure. Click here for file

Additional File 2 **Amino acids classifications based on ribo-seq data only**. The figure is based on ribo-seq profiles which do not include the normalization by mRNA-seq data. Each amino acid was classified as significantly stalling (red), significantly non-stalling (green) or insignificant (black) according to the frequency of its codons in the *USRs*. Stalling amino acids that passed FDR at the 0.05 level are marked with asterisk and those that passed FDR at the 0.1 level are marked by black dots. All analyzed datasets are listed to the left. Thick horizontal white lines are plotted to separate the different organisms. A color bar with the different significance levels is provided to the right. Click here for file

Additional File 3 **The results of a stricter threshold for the sparse data filtering**. The figure is based on RD/mRNA profiles with at least 60% non-zero read counts (see details in the Methods section: *The robustness of the reported results to a stricter threshold of coverage data*). Each amino acid was classified as significantly stalling (red), significantly non-stalling (green) or insignificant (black) according to the frequency of its codons in the *USRs*. Stalling amino acids that passed FDR at the 0.05 level are marked with asterisk and those that passed FDR at the 0.1 level are marked by black dots. All analyzed datasets are listed to the left. Thick horizontal white lines are plotted to separate the different organisms. A color bar with the different significance levels is provided to the right. Click here for file

## Declarations

The publication costs were funded by Tel Aviv University resources.

This article has been published as part of *BMC Genomics *Volume 16 Supplement 10, 2015: Proceedings of the 13th Annual Research in Computational Molecular Biology (RECOMB) Satellite Workshop on Comparative Genomics: Genomics. The full contents of the supplement are available online at http://www.biomedcentral.com/bmcgenomics/supplements/16/S10.
