# Supplementary figures and images for: A comparative genomics study on the effect of individual amino acids on ribosome stalling
Source: BMC Genomics. 2015 Oct 2;16(Suppl 10):S5. doi: 10.1186/1471-2164-16-S10-S5 (PMC4602185; doi:10.1186/1471-2164-16-S10-S5)

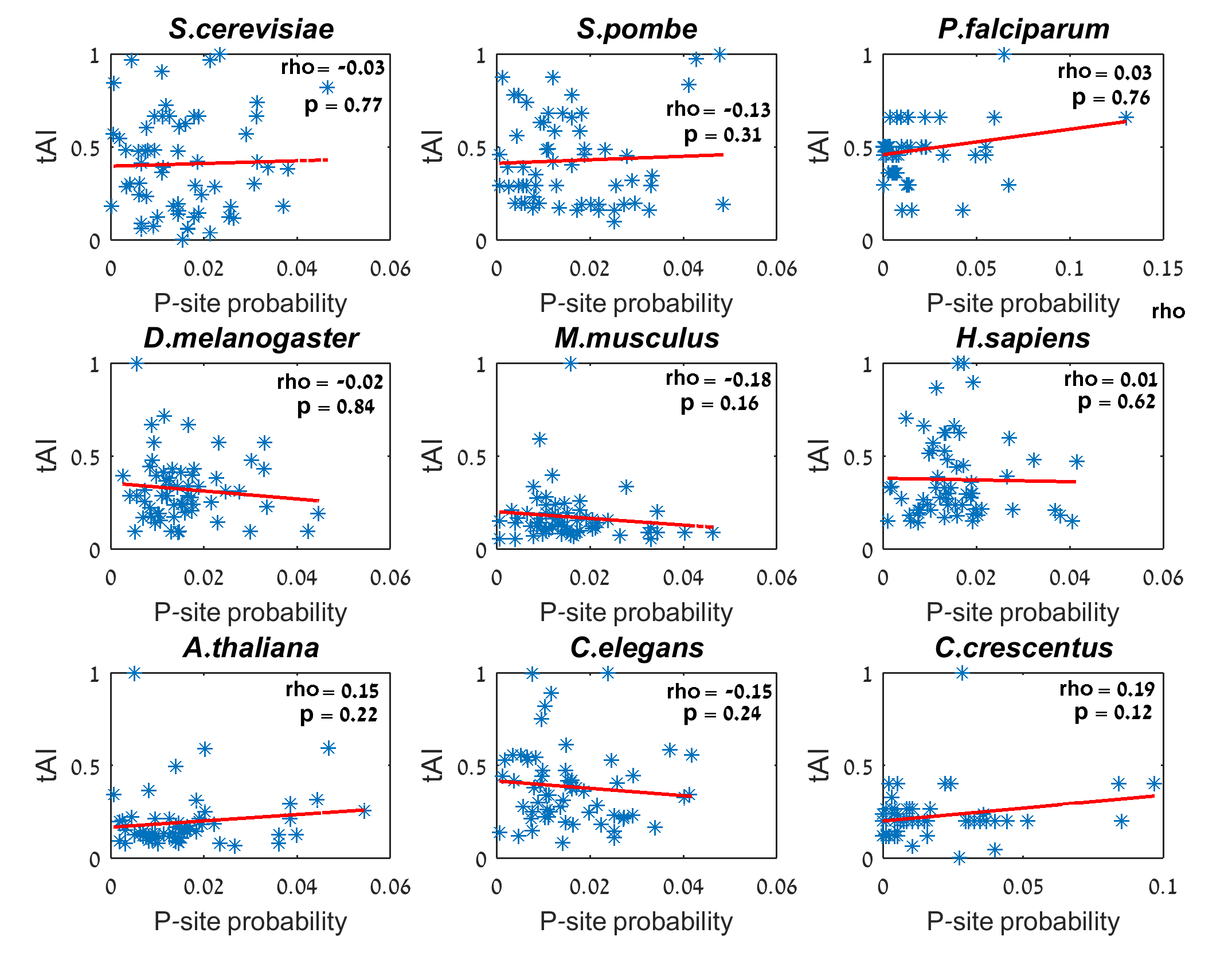

Supplement: Additional File 1 — The correlation between tAI and P-site occupation probability at peak positions. The results are presented per organism based on an aggregate that merges all analyzed datasets of the organism (see details in the Methods section: Merging all datasets of the organism into one aggregate). The probability at the x-axis represents the probability that each of the 61 sense codons occupies the P-site at peak positions. Spearman's rank correlation coefficient (rho) and a corresponding p-value (p) are to the upper right hand corner of each figure. [file 1471-2164-16-S10-S5-S1.png]

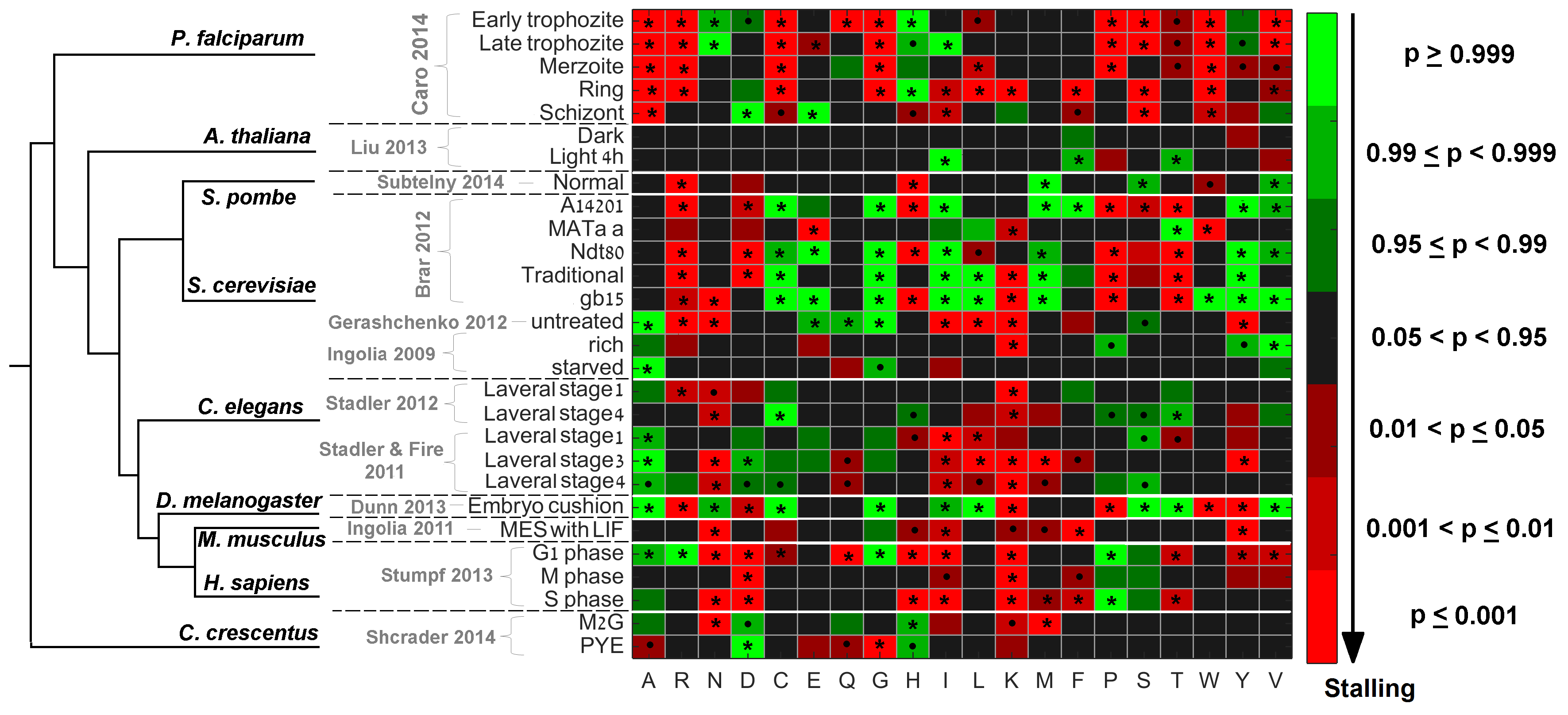

Supplement: Additional File 2 — Amino acids classifications based on ribo-seq data only. The figure is based on ribo-seq profiles which do not include the normalization by mRNA-seq data. Each amino acid was classified as significantly stalling (red), significantly non-stalling (green) or insignificant (black) according to the frequency of its codons in the USRs. Stalling amino acids that passed FDR at the 0.05 level are marked with asterisk and those that passed FDR at the 0.1 level are marked by black dots. All analyzed datasets are listed to the left. Thick horizontal white lines are plotted to separate the different organisms. A color bar with the different significance levels is provided to the right. [file 1471-2164-16-S10-S5-S2.png]

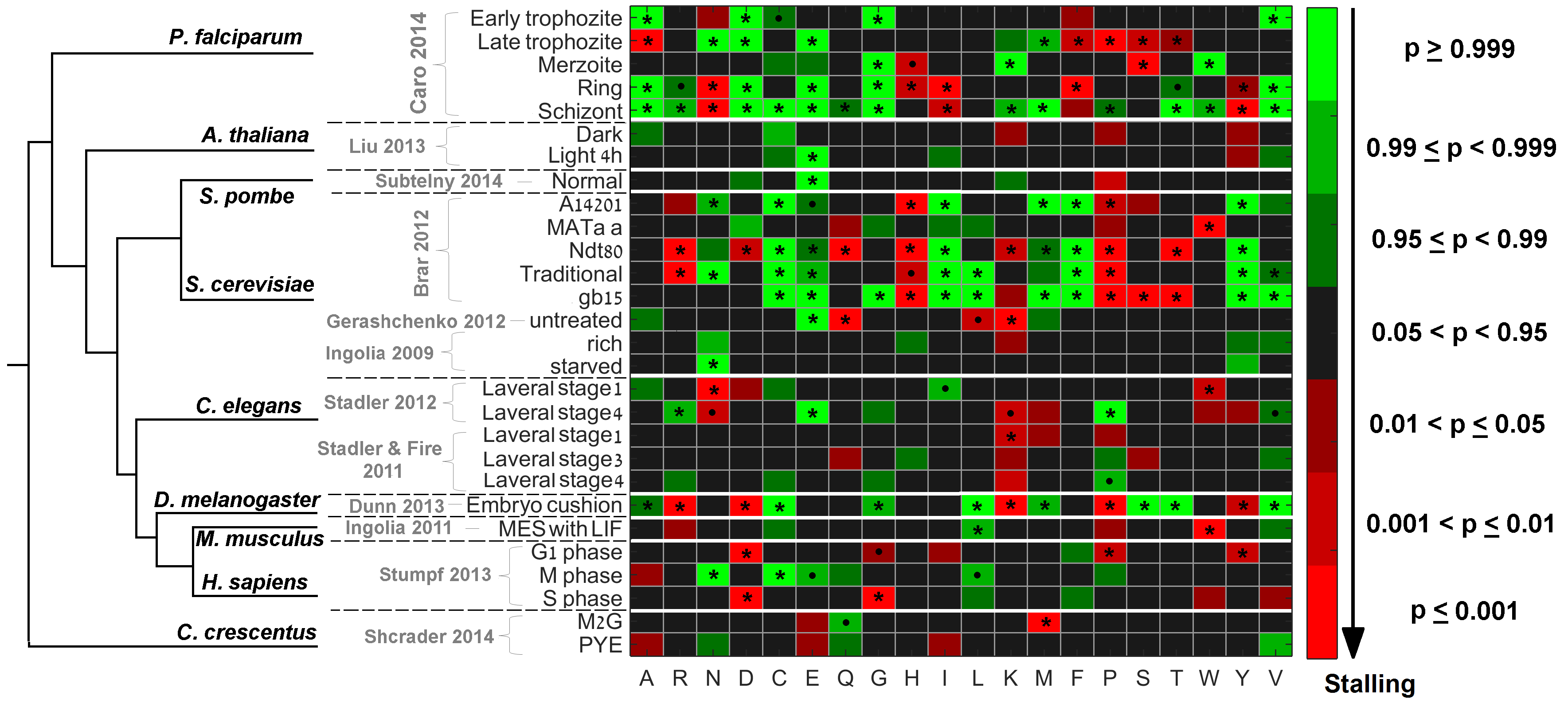

Supplement: Additional File 3 — The results of a stricter threshold for the sparse data filtering. The figure is based on RD/mRNA profiles with at least 60% non-zero read counts (see details in the Methods section: The robustness of the reported results to a stricter threshold of coverage data). Each amino acid was classified as significantly stalling (red), significantly non-stalling (green) or insignificant (black) according to the frequency of its codons in the USRs. Stalling amino acids that passed FDR at the 0.05 level are marked with asterisk and those that passed FDR at the 0.1 level are marked by black dots. All analyzed datasets are listed to the left. Thick horizontal white lines are plotted to separate the different organisms. A color bar with the different significance levels is provided to the right. [file 1471-2164-16-S10-S5-S3.png]
